# Carotid Artery Tortuosity With a Vertebral Artery Entering the Transverse Foramen at an Atypical Level: A Cadaveric Case Underlining the Need for Thorough Anatomical Assessment Before a Stellate Ganglion Block

**DOI:** 10.7759/cureus.97102

**Published:** 2025-11-17

**Authors:** Takutoshi Inoue, Toru Yamamoto, Naotaka Kishimoto, Kenji Seo

**Affiliations:** 1 Department of Anatomy, Teikyo University School of Medicine, Tokyo, JPN; 2 Division of Dental Anesthesiology, Faculty of Dentistry & Graduate School of Medical and Dental Sciences, Niigata University, Niigata, JPN

**Keywords:** cadaver, carotid tortuosity, common carotid, stellate ganglion block, vertebral artery

## Abstract

A stellate ganglion block (SGB) is a therapeutic procedure that transiently interrupts the cervical sympathetic trunk using a local anesthetic, applied not only for pain management but also for conditions such as facial nerve palsy, sudden sensorineural hearing loss, hyperhidrosis, post-traumatic stress disorder, and certain cardiac arrhythmias. The widespread adoption of ultrasound guidance has improved both the safety and the precision of SGB.

During cadaveric dissection at Teikyo University School of Medicine in 2024, a rare coexistence of right common carotid artery (RCCA) tortuosity and a high-rising right vertebral artery (RVA) was identified in an 86-year-old female. The RVA ascended superior to the upper margin of the thyroid gland and likely entered the transverse foramen at C4-C5, in contrast to the left vertebral artery, which entered at the typical C6 level. This previously unreported combination of vascular anomalies underscores the diversity of cervical vascular anatomy and highlights potential risks during neck procedures, including SGB, tracheostomy, thyroid surgery, and anterior cervical spine interventions. Pre-procedural ultrasound provides a noninvasive and cost-effective method for mapping vascular structures; however, its effectiveness depends on the clinician’s anatomical expertise and interpretive accuracy. This case emphasizes the importance of integrating detailed anatomical knowledge with ultrasound assessment to enhance procedural safety and prevent iatrogenic complications.

## Introduction

A stellate ganglion block (SGB) is a therapeutic procedure in which the cervical sympathetic trunk is transiently interrupted by the administration of a local anesthetic [1]. Its clinical applications are broad, extending beyond pain management to include the treatment of facial nerve palsy, atypical facial pain and migraines, sudden sensorineural hearing loss, hyperhidrosis, post-traumatic stress disorder, and certain cardiac arrhythmias [2]. In recent years, ultrasound-guided SGB has become increasingly prevalent. The ability to visualize target structures in real time and enhance the accuracy of needle placement has been shown to reduce the risk of complications such as vascular or soft-tissue injury as compared to traditional landmark-based techniques [3].

From an anatomical perspective, SGB is typically performed at the level of the sixth cervical vertebra (C6), where the prominent anterior tubercle serves as an important landmark [4]. The carotid sheath, containing the common carotid artery, internal jugular vein, and vagus nerve, lies anterolateral to the longus colli muscle while the vertebral artery usually enters the transverse foramen at the C6 level and ascends posteriorly. However, variations in the course or level of entry of the vertebral or carotid arteries are not uncommon and may increase the risk of vascular puncture or intravascular injection during SGB. A detailed understanding of the cervical sonoanatomy and its individual variations is therefore essential for performing this procedure safely and effectively [4].

In the present case, identified during cadaveric dissection, we observed tortuosity of the carotid artery associated with an unusually high-rising vertebral artery entering the transverse foramen above the typical C6 level. This previously unreported combination of vascular anomalies highlights the clinical and educational importance of recognizing cervical vascular variations when planning or performing ultrasound-guided SGB.

## Case presentation

This case report was conducted in accordance with the principles of the Declaration of Helsinki and adhered to the guidelines for cadaveric research established by the Japanese Association of Anatomists [5]. The donor had provided written consent for the use of her body in anatomical education and research.

During an anatomy training session at Teikyo University School of Medicine in 2024, we identified an anatomical variation of the carotid artery. This case was that of an 86-year-old woman, measuring 138 cm in height and weighing 51 kg, with a body mass index (BMI) of 26.0 kg/m². The cause of death was septic shock. Prior to dissection, formalin was injected into the left femoral artery, and the body was preserved under refrigeration with a mixture of alcohol and formalin.

**Figure 1:**
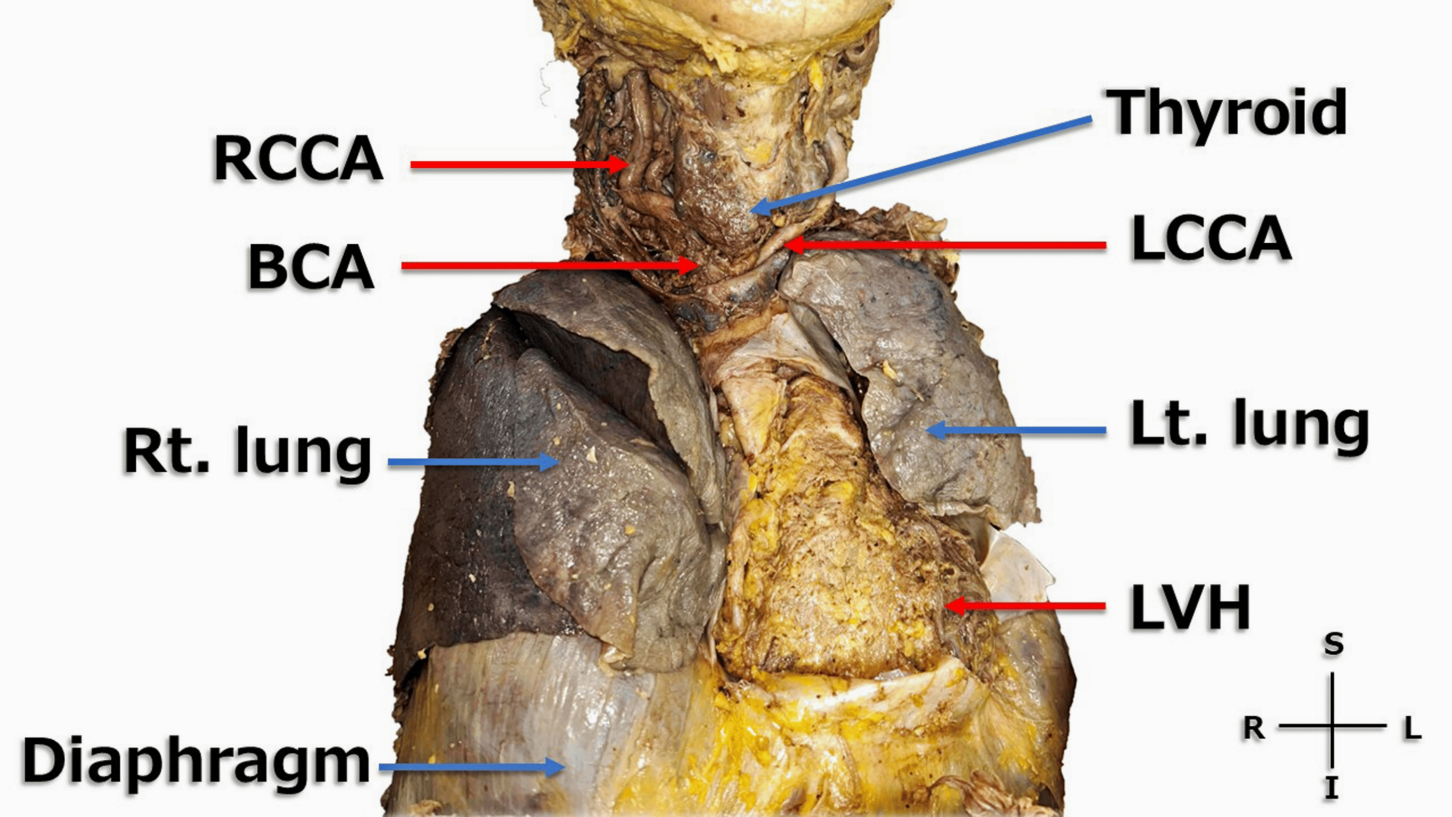
The heart and its surroundings (front) The thoracic cavity was opened, revealing left ventricular hypertrophy (LVH) and a cardiothoracic ratio (CTR) greater than 50%. Subsequently, marked tortuosity of the right common carotid artery (RCCA) was identified. In addition, the brachiocephalic artery (BCA), the left common carotid artery (LCCA), and both the right and left lungs were clearly visualized. Scale reference was not included because quantitative measurements were not feasible in this cadaveric setting. S: superior; I: inferior; R: right; L; left

**Figure 2:**
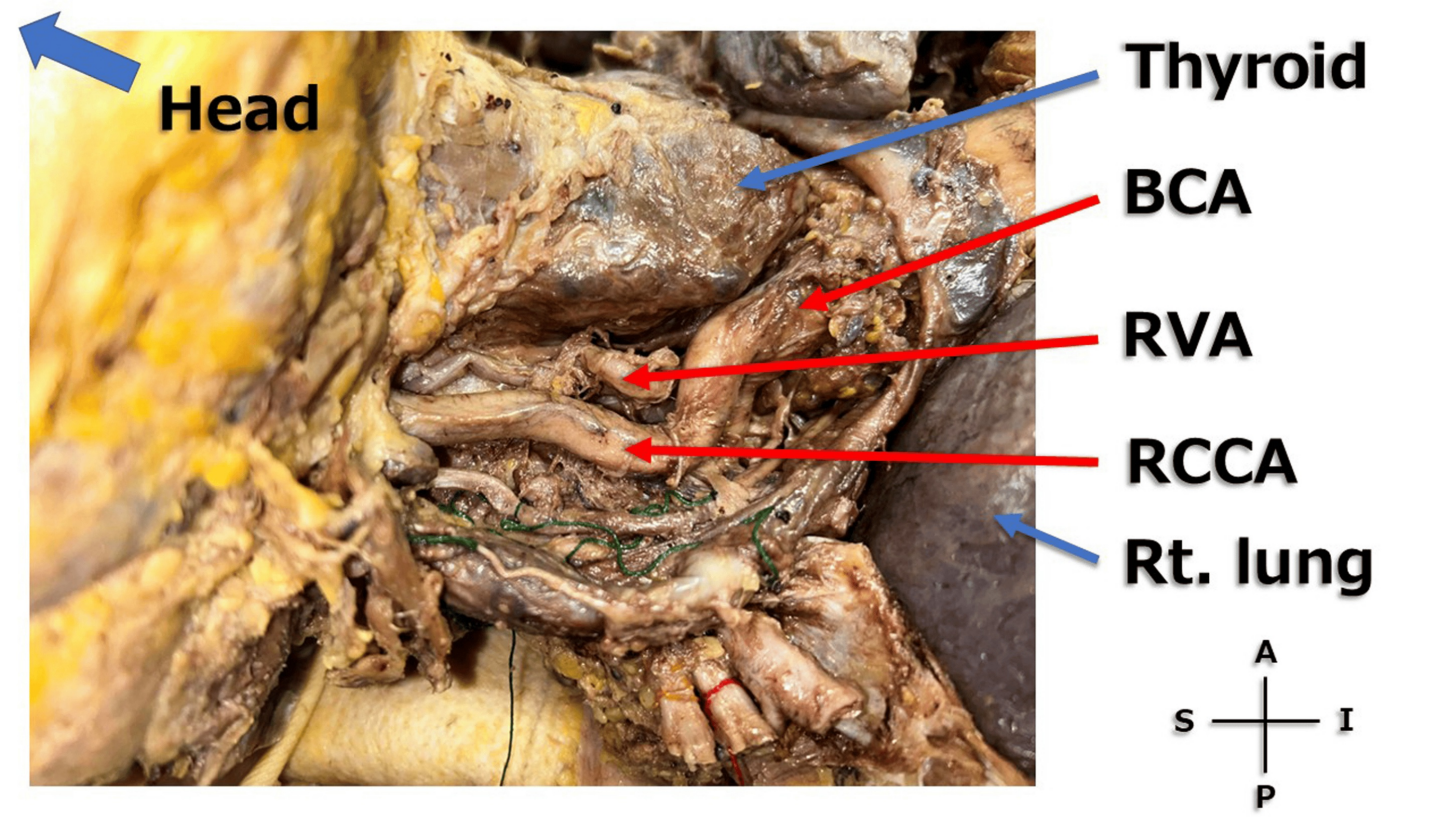
Right neck (right outer surface) A magnified image of the right neck (right outer surface) is shown. Scale reference was not included because quantitative measurements were not feasible in this cadaveric setting. BCA: brachiocephalic artery; RVA: right vertebral artery; RCCA: right common carotid artery; Rt lung: right lung; S: superior; I: inferior; A: anterior; P: posterior

**Figure 3:**
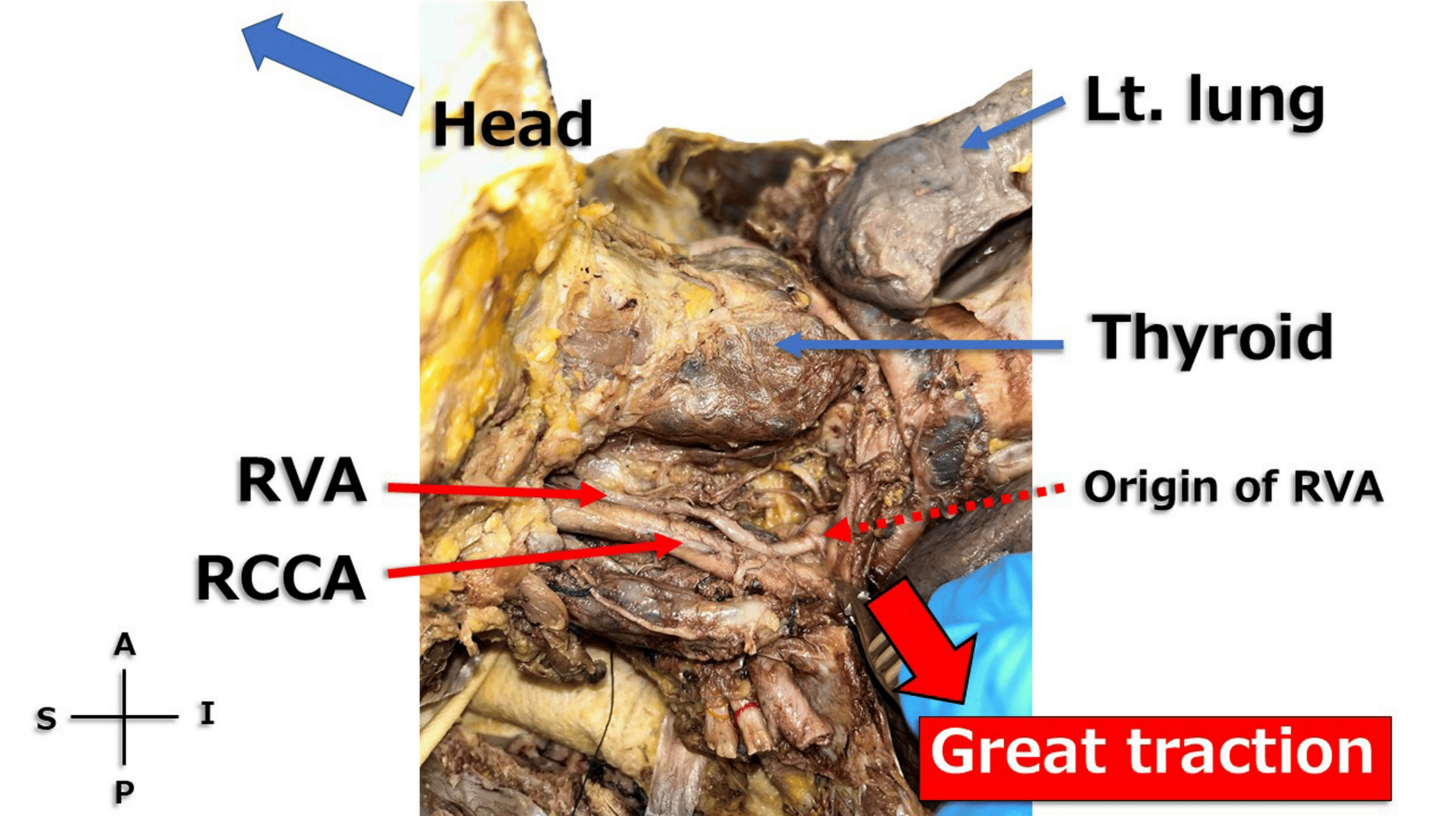
Right neck when towed When the right common carotid artery (RCCA) was gently but firmly retracted laterally, the origin of the right vertebral artery (RVA) became clearly identifiable, and its course was observed to ascend superior to the upper margin of the thyroid gland. Scale reference was not included because quantitative measurements were not feasible in this cadaveric setting. Lt. lung: left lung; S: superior; I: inferior; A: anterior; P: posterior

During the thoracic dissection, marked cardiomegaly and tortuosity of the right common carotid artery (RCCA) were observed (Figure 1). Medial to the RCCA, a tortuous artery ascending cranially was identified and suspected to be the right vertebral artery (RVA) (Figure 2). When the RCCA was retracted laterally, the origin of the RVA arising from the subclavian artery was clearly visualized (Figure 3).

**Figure 4:**
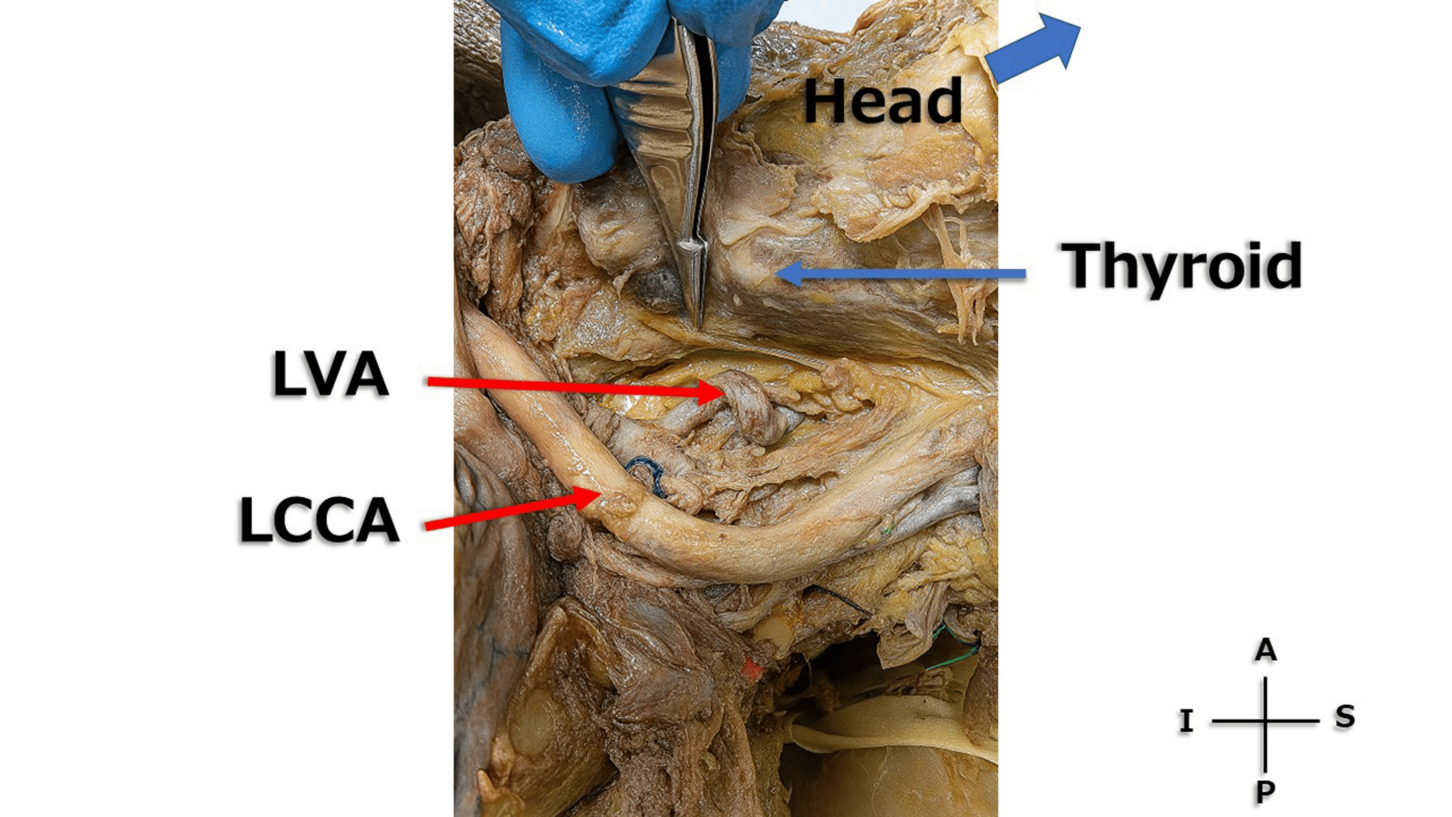
Left neck (left outer surface) A magnified image of the left neck (left outer surface) is shown. The longus colli muscle was not clearly distinguishable in this cadaveric specimen and therefore could not be labeled. Scale reference was not included because quantitative measurements were not feasible in this cadaveric setting. LVA: left vertebral artery; LCCA: left common carotid artery; S: superior; I: inferior; A: anterior; P: posterior

The exact transverse foramen entered by the RVA could not be determined due to partial overlap with surrounding bony structures. However, its course was clearly observed to ascend superior to the upper margin of the thyroid gland. Because the thyroid gland typically extends between the levels of the fifth cervical and the first thoracic vertebrae, this observation suggests that the RVA entered the transverse foramen above C6, most likely around C4-C5. In contrast, the left vertebral artery also exhibited mild tortuosity but entered the transverse foramen at a lower level, approximately corresponding to C6, inferior to the thyroid gland (Figure 4). The longus colli muscle was not clearly distinguishable in this specimen and therefore could not be labeled.

## Discussion

The carotid artery tortuosity observed in this case represents an uncommon vascular anomaly in which the carotid or brachiocephalic artery becomes elongated and twisted, occasionally manifesting clinically as a pulsatile cervical mass [6]. This condition is regarded as an idiopathic anatomical variation and is most often discovered incidentally during head and neck surgery or imaging examinations, while clinical symptoms are rare [7]. It is generally more prevalent in women, with a higher incidence on the right side [8]. Reported predisposing factors include advanced age, obesity, atherosclerosis, hypertension, and cardiomegaly [8]. In this case, the donor was an elderly woman with cardiomegaly and a body mass index (BMI) of 26.0 kg/m², corresponding to an overweight status, which may have contributed to vascular elongation and tortuosity [9].

In addition, the RVA in this case was found to course superior to the thyroid gland and to enter a transverse foramen at a different level from the left vertebral artery. Typically, the thyroid gland is located between the levels of the fifth cervical and the first thoracic vertebrae [10]. In this cadaver, the RVA ascended beyond the upper margin of the thyroid gland and likely entered the transverse foramen at the level of either the fourth or fifth cervical vertebra. A recently published systematic review and meta-analysis reported that most vertebral arteries enter the transverse foramen at the C6 level, but recent anatomical studies have demonstrated considerable variability in vertebral artery pathways, including high-entry variants and the presence of accessory transverse foramina, which may influence the course of the artery and its susceptibility to iatrogenic injury [11,12]. Such deviations are thought to arise during embryological development, when abnormal persistence or atypical fusion of intersegmental arteries leads to variant vertebral artery pathways. The concurrent occurrence of carotid tortuosity and a high-entry vertebral artery is rare, as these anomalies usually develop independently; this combination may indicate a shared embryologic disturbance or age-related vascular remodeling.

An SGB is most commonly performed by pain specialists and anesthesiologists. In patients with hypertension who present with a palpable pulsation in the right supraclavicular fossa, tortuosity of the right common carotid artery should be suspected, warranting thorough pre-procedural evaluation [6]. Furthermore, as in the present case, the possibility of a tortuous vertebral artery entering the transverse foramen at an atypical level must be considered. Pre-procedural ultrasound imaging should therefore be employed. Ultrasound provides clear visualization without radiation exposure and at relatively low cost, making it an ideal modality for preoperative assessment in SGB [13]. It allows direct identification of anatomical variations prior to needle insertion, facilitates the planning of the needle trajectory, and minimizes the risk of injury to adjacent structures. During the procedure, real-time ultrasound guidance further reduces the likelihood of complications such as local anesthetic toxicity or recurrent laryngeal nerve palsy [13]. In addition, the ability to digitally record obtained images provides valuable resources for postoperative review and educational purposes. Beyond SGB, awareness of such vascular variations is equally crucial during tracheostomy, thyroidectomy, cervical lymph node dissection, and anterior cervical spine surgery, where unrecognized arterial anomalies may lead to catastrophic bleeding.

This case further underscores several key learning points relevant to both anatomists and clinicians: (1) cervical vascular variations are more common than generally appreciated and may coexist in the same individual; (2) ultrasound imaging should be integrated into pre-procedural planning to map vascular anatomy; and (3) procedural safety ultimately depends on the clinician’s ability to interpret sonographic findings in the context of detailed anatomical knowledge.

We recommend that the target of the SGB first be identified at the C6 level, and that the ultrasound probe then be scanned cranially and caudally to locate the vertebral artery. Once the vertebral artery is identified, its course should be followed to the point where it enters the cervical transverse foramen to ensure that it does not lie within the intended needle trajectory. This systematic scanning approach enhances procedural safety and allows early recognition of atypical vertebral artery pathways that could otherwise increase the risk of iatrogenic injury.

A limitation of this report is the lack of radiological or histological confirmation of the vascular course, as well as the absence of quantitative measurements due to the nature of cadaveric observation. Nevertheless, direct anatomical inspection provided clear macroscopic evidence of a rare combination of vascular anomalies with substantial clinical relevance.

## Conclusions

In this cadaveric case, tortuosity of the right common carotid artery was observed in association with a tortuous right vertebral artery entering the transverse foramen at an atypically high cervical level. This rare coexistence highlights the diversity of cervical vascular anatomy and the potential risk it poses during procedures involving the neck, such as stellate ganglion block, tracheostomy, thyroid surgery, or anterior cervical spine interventions. Ultrasound imaging serves as an essential, noninvasive tool for detecting such variations, but accurate interpretation requires solid anatomical understanding. Safe and reliable performance of SGB and other cervical interventions demands preoperative awareness of vascular anomalies, particularly variations in the carotid and vertebral arteries, and integration of anatomical knowledge with ultrasound-based assessment to prevent iatrogenic complications.
